# Why does the mind wander?

**DOI:** 10.1093/nc/niz014

**Published:** 2019-10-22

**Authors:** Joshua Shepherd

**Affiliations:** 1 Department of Philosophy, Carleton University, 3A61 Paterson Hall, 1125 Colonel By Drive, Ottawa, ON K1S5B6, Canada; 2 Faculty of Philosophy, Universität de Barcelona, Carrer Montalegre 6, Barcelona, 08001, Spain

**Keywords:** mind wandering, cognitive control, agency, intention, volition

## Abstract

I seek an explanation for the etiology and the function of mind wandering episodes. My proposal—which I call the cognitive control proposal—is that mind wandering is a form of non-conscious guidance due to cognitive control. When the agent’s current goal is deemed insufficiently rewarding, the cognitive control system initiates a search for a new, more rewarding goal. This search is the process of unintentional mind wandering. After developing the proposal, and relating it to the literature on mind wandering and on cognitive control, I discuss explanations the proposal affords, testable predictions the proposal makes, and philosophical implications the proposal has.

**Highlights**

- Makes a novel and empirically tractable proposal regarding why the mind wanders
- Offers novel explanations of data on mind wandering
- Offers predictions for future work on mind wandering
- Integrates literature on cognitive control with the literature on mind wandering
- Discusses implications for a philosophical account of the nature of mind wandering

## Introduction

### Minds wander

Some wander more than others, but human ones wander a lot. A much-cited estimate, due to Killingsworth and Gilbert (2010), has it that the awake human mind spends from a third to half its time wandering. That’s a big range, a rough estimate, and there are good reasons to be suspicious of it (see Seli *et al.* 2018). The actual number will likely depend a bit upon the nature of mind wandering, a bit upon whether we have the right measure to produce such an estimate, and of course a bit on individual variability. Estimates aside, though, introspection reports that the mind wanders surprisingly often. My question here is this.

### Why does it happen?

Sub-questions include the following. What drives the mind to wander? Does anything drive it to wander? Is the transition from focused thought to meandering thought random? Is it a failure of control, or is there some dark purpose behind these mental movements?

In the next section, I set the table by discussing a few interesting features of mind wandering, as well as a few recent proposals about its etiology, and its function. It is easy to conflate these two, since if mind wandering has a function its etiology may very well help illuminate it, but the questions are distinct. Here, I am more interested in why mind wandering happens—about its etiology. It turns out, though, that on my proposal mind wandering happens for good functional reasons. I develop this proposal, which I call the cognitive control proposal, in Cognitive control and Why the mind wanders sections. In Explanations section, I discuss some explanations this proposal makes possible. In Predictions section, I discuss some predictions that could confirm or disconfirm the proposal. In Philosophical implications section, I discuss implications for a philosophical account of the nature of mind wandering.

## Mind Wandering

The term “mind wandering,” used as a construct in psychological theorizing, is relatively young. In a seminal paper, Smallwood and Schooler (2006) argued that research on disparate constructs—they mention task-unrelated thought, task-unrelated images, stimulus-independent thought, mind pops, and zone outs—could be unified under a single moniker.

By referring to this phenomenon as mind wandering, a term familiar to the lay person, we hope to elevate the status of this research into mainstream psychological thinking (946).

As Murray *et al.* (2019) report, since that review, usage of “mind wandering” has risen dramatically. Only the Smallwood and Schooler paper used the term in a title or abstract in 2006. In 2018, the term appeared in 132 titles or abstracts.

Increased attention to the range of phenomena grouped together by “mind wandering” is salutary. But theorists recognize that the range of processes the term groups may contain multiple etiologies and processing signatures. Accordingly, theorists have proposed many sub-types of mind wandering, categorizing episodes of mind wandering in at least three distinct ways.

The first two involve a conception of mind wandering as defined in part by the contents of a mind-wandering episode, where the contents are unrelated to a task an agent was performing, or was supposed to perform. But there are various ways for an agent to engage in task-unrelated thought. Some categorize mind-wandering episodes in terms of a relationship to an agent’s intention: mind wandering might occur intentionally or unintentionally (Giambra 1995; Seli *et al.* 2016). A second way to categorize mind-wandering episodes is in terms of a relationship to external stimuli. One might here distinguish between distraction, when the mind is prompted to wander by external stimuli, and mind wandering, when the mind is prompted to wander by internal processes, independently of any particular stimuli (see Stawarczyk *et al.* 2013). Or one could argue that distraction, especially sustained distraction, is a legitimate mind wandering as well.

A third way to characterize mind wandering is not in terms of its contents, but rather its dynamics. So, e.g., Christoff *et al.* (2016) characterize mind wandering as a species of spontaneous thought, with distinct dynamics. Mind wandering is distinguished from creative thought, and rumination, and other types of mental episodes, by relation to the presence or absence of various constraints on the episode (e.g., what they call “deliberate” and “automatic” constraints).

From a certain height, it appears that these different characterizations may not be in competition. Perhaps there are many routes to mind wandering. Perhaps some of them overlap. Perhaps different questions can be answered by focusing on certain routes in certain contexts. Reasonably, Seli *et al.* (2018) have recently argued in favor of mind wandering as a natural kind, with different sub-types grouped together by relations akin to family resemblance: “We propose that the field acknowledge mind-wandering to be a multidimensional and fuzzy construct encompassing a family of experiences with common and unique features” (2018, 482).

Thinking of mind wandering in this way fits well with other work on the nature of natural kinds, according to which tokens of a natural kind share some—but not necessarily all—of a class of stable similarities, which cluster together thanks to underlying causal mechanisms (Boyd 1991). Thinking of mind wandering in this way does not foreclose the possibility that further empirical exploration will reveal important distinctions amongst sub-types of mind wandering, or will reveal a need to revise the construct of mind wandering in favor of a construct (or constructs) more precise or fruitful (for a similar point, and criticism of the family resemblances view, see Christoff *et al.* 2018). Nor does thinking of mind wandering in this way remove the need for precision regarding the sub-types of mind wandering. Seli *et al.* (2018), e.g., advise that papers on mind wandering remain explicit, as follows.

Methodological and conceptual clarity will simply require, in empirical manuscripts, something like the following sentence: “Here, we conceptualized mind-wandering as ________, and operationally defined it for our participants as ________.” Critically, this approach allows researchers the freedom to study whatever features of mind-wandering they wish, while providing the required specificity about aspects of the experience being explored. (488)

In the same spirit, I note here the sub-type of mind wandering that concerns me. I am interested in unintentional mind wandering—episodes of mind wandering that are neither initiated nor governed by any reportable intention of the agent. This category may cross-cut any relationship to external stimuli, in the sense that unintentional mind wandering could be externally or internally initiated. And it may demonstrate dynamics that are distinct from other sub-types of mind wandering.

Unintentional mind wandering could in principle happen non-consciously. But the literature on human mind wandering has it pegged as a feature of the conscious mind. That is to say, when the mind wanders, what wanders is the stream of consciousness—processes of conscious mentation. So, one key way to study mind wandering is to ask people whether or how often their mind has wandered. People offer reports about it. They recognize that they have been mind wandering. This is not because of mind wandering’s phenomenological signature. It is rather because people have a sense that they were once up to something, and then, more or less unbeknownst to them, they began to be up to something else. Thomas Metzinger (2013) speaks of this as the self-representational blink: an unnoticed shift from pursuing one task to doing whatever it is we do when the mind wanders. Recognizing that your mind has been wandering is always slightly surprising, because you did not plan for things to go in that way. From your perspective, it seems that *they just did*.

This is puzzling. But calling a mental episode unintentional need not imply that mind wandering is maladaptive, or that it has no function. Indeed, the very frequency with which it occurs had led many to suggest that it must have some functional role (e.g., Baird *et al.* 2011). It may not, of course. Perhaps, we survive in spite of how mentally addled we all are. But it is at least plausible that there is a function.

Some accounts of mind wandering might be taken to deny this. McVay and Kane (2010a) and Kane and McVay (2012), e.g., have argued that mind wandering reflects a failure of executive control. They note that a negative correlation exists between working memory capacity and a tendency to experience task-unrelated thoughts (see also Randall *et al.* 2014). Some such correlation is plausible. When one experiences task-unrelated mentation, something has clearly gone wrong. One has failed to stay on task.

But this also fails to imply that mind wandering has no function. Kane and McVay note that the correlation between working memory capacity and task-unrelated thought is not terribly strong: “WMC accounts for only about 5% of the variability in [task-unrelated thought] TUT rates (and vice versa)” (2012, 352). It is possible that mind wandering is both a failure in one sense and adaptive in another.

Indeed Kane and McVay’s data indicate this in the following way. They demonstrate that when task demands are high, greater working memory capacity is correlated with fewer task-unrelated thoughts. Other work suggests that when task demands are low, greater working memory capacity correlates with the occurrence of more task-unrelated thoughts (Levinson *et al.* 2012), although a preregistered replication attempt failed to find the same correlation (Meier 2019). Further, recent work demonstrates that the role of cognitive control in mind wandering is somewhat malleable, with those who possess more cognitive control mind wandering at more strategic times (Seli *et al.* 2018). Rummel and Boywitt (2014) found that participants with higher working memory capacity both [a] demonstrated an increased tendency to mind wander (operationalized as the experience of task-unrelated thoughts (TUTs)) in less demanding tasks, and [b] showed less performance decrements on these same tasks. They comment:

[W]e found evidence for the hypothesis that cognitive control abilities are specifically involved in the flexible adjustment of mind-wandering to task demands. As was hypothesized, high-WMC participants showed higher levels of TUT adjustment than did low-WMC participants. Thus, a more flexible coordination of the stream of thought appears to be characteristic of high-WMC individuals: They engage in TUTs when situational demands are low but reduce TUTs in attention-demanding situations. (1313)

This hypothesis is consistent with work that has demonstrated that as cognitive control resources diminish with age, the propensity to mind wander diminishes as well (Maillet and Schacter 2016).

If we are to believe that mind wandering is associated with deployments of cognitive control, we need evidence that when agents mind wander, they engage in thought processes that may be beneficial. Some evidence for this is that when agents mind wander, their thoughts very frequently go to non-occurrent goals and needs, and to mentation about how to satisfy these goals in the future (Klinger 1999; Baird *et al.* 2011).

Indeed, as Irving and Thompson (2019) note, it seems that it is possible to manipulate the content of mind wandering episodes by giving agents specific goals. Morsella *et al.* (2010) told some participants they would, in the near future, have to answer questions about the states in America. Then they gave the participants a different task. About 70 percent of these participants’ task-unrelated thoughts were about U.S. geography. Similarly, Mac Giolla *et al.* (2017) gave some participants a real future task, and told different participants to only pretend to have (or to lie about having) the same future task. Those participants with genuine intentions reported much more spontaneous thought about the future task than participants without genuine intentions.

It is also possible to manipulate mind wandering by reminding agents of their goals. Kopp *et al.* (2015) had participants either construct a list of their plans for the week or list features of a car. Participants then performed a reading task. Participants who had just reviewed a set of their own plans and goals reported much more mind wandering during the reading.

There is thus an apparent tension within mind wandering. When the mind wanders (at least unintentionally), agents are distracted from the current task, and performance suffers. But when the mind wanders, it tends to find non-occurrent goals the agent possesses, generating planning that could be beneficial. What’s more, greater cognitive control is associated with increases in mind wandering, especially when task demands are low.

Recall my original question: why does the mind wander? Two related questions that could help: What causes it to start, and what explains what happens as it wanders?

My proposed answer runs through recent work on cognitive control, and on what kinds of mechanisms drive allocations of cognitive control resources. I discuss this work in the next section.

## Cognitive Control

Although the term “cognitive control” is in wide use in psychology and neuroscience, pinning down its referent is not straightforward. In an influential paper, Botvinick *et al.* characterize it in terms of flexible adjustment of processing towards some end (or set of ends):

A remarkable feature of the human cognitive system is its ability to configure itself for the performance of specific tasks through appropriate adjustments in perceptual selection, response biasing and the on-line maintenance of contextual information. The processes behind such adaptability, referred to collectively as cognitive control … (Botvinick *et al.* 2001, 624)

Rouault and Koechlin likewise emphasize processes of regulation towards certain ends: “Cognitive control refers to mental processes that evolve as regulating adaptive behavior beyond basic reinforcement and associative learning processes” (2018, 106).

There is a danger here, analogous to the one just discussed regarding definitions of mind wandering, in including far too many process-types under the same heading. “Cognitive control” includes processes like the construction and maintenance of a task set, the switching from one task set to another, the deployment of attention in various ways, the deployment of inhibition, and the monitoring of an agent’s progress towards goal achievement. To get better at understanding how these processes work together (or don’t), it helps to have a label. But the nature of the system is only loosely delineated.

Given this, there is room for differing emphases. So, e.g., Adele Diamond characterizes cognitive control processes as “a family of top-down mental processes needed when you have to concentrate and pay attention, when going on automatic or relying on instinct or intuition would be ill-advised, insufficient, or impossible” (136). This characterization is useful, but not definitive. For the kind of cognitive control processes, I have in mind here might be considered top-down, but do not activate only when agents need to deploy attention. These processes operate outside of the agent’s awareness, influencing the agent’s thought and action in subtle and difficult to detect ways.

So, e.g., Kurzban *et al.* (2013) have argued that one subtle way cognitive control mechanisms influence thought and action is by generating an experience of effort related to the performance of some task. They hypothesize that the experience of effort is the result of sub-personal computations that determine the current task’s value, as well as the value of nearby available tasks, and output a determination of the opportunity cost of persisting on the current task. The experience of effort is hypothesized to be a signal to the agent to switch tasks.

Kurzban *et al.*’s proposal has received a lot of attention. Few agree with all of the specifics, but most agree with the general perspective that sub-personal monitoring mechanisms are concerned to determine the value of succeeding in the current task, as well as the cost of continuing engagement in the current task, and are concerned to, in some sense, direct the agent or her cognitive control resources in a more fruitful way.

Perhaps the most mature theory characterizing the mechanisms that constitute the allocation of cognitive control is the Expected Value of Control theory (see Shenhav *et al.* 2013, 2017). The general idea is that the cognitive control system “specifies how much control to exert according to a rational cost-benefit analysis, weighing these effort costs against attendant rewards for achieving one’s goals” (Lieder *et al.* 2018, 2). Lieder *et al.* add to this idea a sophisticated model of how the cognitive control system might come to learn the value of the various control signals it can deploy, and might rely upon what it learns to guide cognition in adaptive ways.

Lieder *et al.* characterize the position the cognitive control system is typically in as a Markov decision process, specified over certain parameters, driven by reinforcement learning. Those parameters are the initial state of the system, the set of states the system could be in, the set of possible actions (or moves, or operations) the system could take, the conditional probabilities of transitioning between states, and a reward function. Lieder *et al.* further characterize the actions the system could take as “control signals that specify which computations the controlled systems should perform” (4).

Given this setup, the main aim is to maximize reward via the specification of control signals. The way the system does this is by way of learning algorithms. The system builds and updates a model that specifies transition probabilities between states given different control signals, and that maps these probabilities onto a reward function. The reward function balances the reward associated with an outcome (a new state), together with the computational costs of specifying the computation required to drive the system towards the outcome. So, what the system is designed to do is to take the action (specify the control signal or the package of control signals) that has the highest expected value, given the probabilities of where the action takes the system, and the costs of taking the action.

The hypothesis here is that “the cognitive control system learns to predict the context-dependent value of alternative control signals” (5), and that these predictions determine which actions the system takes.

In cases in which the context is relatively well-known, Lieder *et al.* posit that the system will depend upon relationships between features of the internal state of the agent and features of the context, and will perform weighted sum calculations to determine the value of various possible actions. Cases in which the context is not well-known are more difficult. But Lieder *et al.* propose that in such cases the system may utilize exploration strategies to teach itself the value of various actions in the novel situation. These exploration strategies involve drawing samples of the value of control signals in previously encountered contexts, averaging over them, and again selecting the control signal that provides the highest expected value.

Lieder *et al.* note that “This model is very general and can be applied to model cognitive control of many different processes” (6). And they offer a proof of concept for it, by demonstrating that their model outperforms alternative models across a range of processing types.

These processing types involve learning what features of a task are predictive of reward. Some of them are quite simple. One task on which their model performed well-involved learning where to allocate attention, based upon variable reward offered for attending to different locations. A second task involved learning the difference between colors that indicate reward, and colors that do not. That the model predicts basic learning of this sort is good, but not too surprising.

Other processing types are more complicated. One task on which the model did well was an incongruent Stroop task run by Braem *et al.* (2012). In this task, participants saw a central square flanked by two other squares. They had to name the color of the square. In one condition, the flankers were the same color as the central square. In another condition, the flankers were incongruently colored. When participants received a reward for their response on a congruent trial, they became much quicker to respond on a subsequent trial, suggesting they had learned that the automatic (low-control) mode of response was best. When participants received a reward for their response on an incongruent trial, they became much slower to respond on a subsequent trial, suggesting they had learned that the high-control mode of response was best. Lieder *et al.* comment:

The expected value of computation depends not only on the rewards for correct performance but also on the difficulty of the task. In easy situations, such as the congruent trials of the Stroop task, the automatic response can be as accurate, faster, and less costly than the controlled response. In cases like this, the expected value of exerting control is less than the EVOC of exerting no control. By contrast, in more challenging situations, such as incongruent Stroop trials, the controlled process is more accurate and therefore has a positive EVOC as long as accurate performance is sufficiently important. Therefore, on incongruent trials the expected value of control is larger than the EVOC of exerting no control. Our model thus learns to exert control on incongruent trials but not on congruent trials. Our model achieves this by learning to predict the EVOC from features of the stimuli. This predicts that people should learn to exert more control when they encounter a stimulus feature (such as a color or word) that is predictive of incongruence than when they encounter a feature that is predictive of congruence. (19)

Of course, agents are rarely aware that a system (or coordinated collection of mechanisms) within them is busy learning the value of different modes of responding, and guiding the way that they deploy cognitive control resources. We are not here explaining explicit deliberation or planning. But we are getting insight into the processes—sub-personal, if you like—that create the cognitive ocean in which more explicit processes swim. What’s more, we are getting insight into the kinds of learning that drive cognitive control operations that agents have to simply live with. Shifts of attention, pulls to engage in various computational operations, a sense of what operations are valuable in what contexts—these are rarely things we explicitly consider. Rather, we depend upon this background to engage in explicit cognition and intentional action.

With this as background, I can suggest an interesting possibility, leading to a proposal regarding the etiology and function of mind wandering.

## Why the Mind Wanders

The possibility is this. Depending on the cognitive control system’s model of the value of various control signals, in cases containing relatively little expected value the system may select a package of control signals leading to exploration. These would be cases in which the goal is to find a new and better goal. And the method, which remains here unclear—although one could imagine it involving shifts of attention, construction of task sets involving imagination, inhibition of current goals, etc.—might be generally described as disengagement from the present task in order to set out upon a search for a more valuable task.

The cognitive control proposal, then, is this. Mind wandering is caused by the cognitive control system precisely when, and because, the expected value of whatever the agent is doing—usually, exercising control towards the achievement of some occurrent goal—is deemed too low, and this “too low” judgment generates a search for a better goal, or task. Perhaps, e.g., the estimation of expected value dips below a value threshold attached to the package of control signals that generate exploration for another goal, or task. Or perhaps the value is always computed in comparison with available options, such that mind wandering is sometimes initiated even in the face of a rewarding current task.

This is a straightforwardly empirical proposal, and should be assessed in terms of the explanations it affords, and by whether the predictions it makes are confirmed or disconfirmed. Before I discuss explanation and prediction, however, I wish to note two things.

First, it would certainly be useful if the cognitive control system contained such an operation. Humans are sophisticated agents, with multiple needs and goals potentially in play in most waking life situations. Fixation on one goal alone, or working towards the satisfaction of one goal at a time, is not a great strategy for flourishing. For, first, if one gets stuck on a difficult goal, or if it becomes apparent (i.e. apparent at least to some system tasked with calculating such a thing) that the present goal is not as rewarding as once calculated, it is much wiser to disengage and seek a better goal. And, second, in many situations progress towards multiple goals at once is possible. All one needs is the capacity to divide attention somewhat, or the capacity to hold multiple goals in mind—or at least within some accessible place—and one might waste much less time. Notice, further, that the above points may hold even if dividing the mind amongst multiple goals leads to performance decrements. Perfect performance is not always required. So long as mediocre performance allows one to satisfy goals and needs, accepting mediocre performance will be a good strategy.

Second, explicit cognitive control already does contain such an operation. Sometimes a task becomes too effortful, too uncomfortable, or too boring. Sometimes—after one has just awakened from a long nap, e.g.—there’s no obvious task at hand. In such cases performing a search for a high-value goal is a familiar operation that we perform explicitly. In other cases, we do not leave behind the current task, but we rather utilize deliberation, prospection, imagination, and other processes in order to find sub-goals, or means to achieve the goal that is currently structuring behavior. These modes of exploration towards discovery of a high-value goal are explicit. Our question here is whether the cognitive control system implicitly—i.e., in the absence of an explicit or conscious formation of intention to do so—initiates mind wandering as a similar mode of exploration, and for similar reasons. The proposal is that it does.

## Explanations

Here are explanations this proposal affords.

First, this proposal offers an explanation for the initiation of mind wandering episodes. These episodes are initiated without the agent’s explicit consent. But they do frequently occur. One possible explanation is that the agent necessarily loses control in these instances. That characterizes the initiation of a mind wandering episode as random. A better explanation, I submit, is that while the initiation of a mind wandering episode is, in one sense, a failure—a failure of the current goal and task set to persist—it is, in another sense, a smart move. It is smart because it results from a cognitive control system that is more or less constantly attempting to determine the value of selecting packages of control signals, and that will act when discrepancies in value are calculated. Note, incidentally, that this could be extended to cases in which the agent is pursuing no particular goal, or has no current task. The system need not always compare value between goals. It might be useful, e.g., to tag expected levels of reward to particular environments, perhaps by averaging over the kinds of rewards an environment-type provides. If agents associate one type of environment—a party, e.g.,—to a plethora of rewarding experiences, then a signal that this environment is near—one can hear party music, e.g.,—might lead the mind to wander in the direction of the kinds of experiences the rewarding environment provides.

The fact that the initiation of mind wandering episodes is smart helps to additionally explain a second fact, namely, that agents with higher levels of cognitive control mind wander more frequently when the current task is easy or non-rewarding.

This is not to deny that mind wandering episodes may sometimes be initiated by affectively salient stimuli, or other distractors. Nor is it to deny the existence of completely unguided, or otherwise guided, episodes of mind wandering. I am not in a position to deny that, e.g., a case of spreading activation in a semantic network could qualify as unintentional mind wandering. It may very well be—indeed it seems plausible—that only some cases of unintentional mind wandering are controlled in the way I here propose. Note, however, that even if this is right, the cognitive control system may be able to interact with uncontrolled mind wandering processes. In some cases, uncontrolled mind wandering could be commandeered if a valuable goal suggests itself.

Third, this proposal offers an explanation for the fact that mind wandering episodes tend to go to other goals the agent possesses. This is a natural place for a process to go if that process is structured by an aim to find a more rewarding goal than the one from which the agent has just disengaged. For it will be much more cost-effective to find existent goals, perhaps by querying memory, than to explore the environment and to construct entirely new goals (although of course this may happen, especially when the environment easily affords novel and rewarding goals).

Fourth, this proposal might be integrated with extant explanations of aspects of mind wandering. Consider, e.g., the decoupling hypothesis (Antrobus *et al.* 1970; Smallwood *et al.* 2003; Smallwood and Schooler 2006)—the idea that once mind wandering is underway, domain-general cognitive processes are engaged to maintain the mind wandering episode, by keeping attention decoupled from perceptual input, and by aiding the “continuity and integrity” of the agent’s train of thought (Smallwood 2013, 524). As Smallwood (2013) notes, the decoupling hypothesis does not seek to explain the initiation of mind wandering. The cognitive control proposal is consistent with it. That is, the proposal is consistent with domain-general resources being deployed to assist mind wandering episodes. The main comment I wish to make here is that the decoupling hypothesis becomes more plausible, and data on the deployment of domain-general resources in mind wandering more transparent, if the entire process of mind wandering can be seen as goal-directed, where the goal is set by the cognitive control system.

This proposal is also consistent with work on the recruitment of neural areas during mind wandering. Christoff *et al.*, e.g. (Christoff *et al.* 2009; Fox *et al.* 2015), have found that episodes of mind wandering recruited not only core areas of the default mode network—medial PFC, posterior cingulate/precuneus, and posterior temporoparietal cortex—but also dorsal anterior cingulate cortex and dorsolateral prefrontal cortex, “the 2 main regions of the executive network” (Christoff *et al.* 2009, 8722). Christoff *et al.* plausibly link the involvement of the executive network with task performance decrements. The cognitive control proposal adds the possibility that executive network recruitment is associated with the goal-directed nature of (at least some) unintentional mind wandering.

Consider, further, recent work on the dynamics of mind wandering. In a recent review, Christoff *et al.* (2016) rightly notice that much research on mind wandering has been content-based, “assessing the contents of thoughts in terms of their relationship to an ongoing task or activity” (722). They seek, instead, to offer a taxonomy of thought-types in terms of their dynamics—of how they operate over time. They propose two dimensions along which the dynamics of thought may be influenced. The first dimension is characterized in terms of the degree to which thought is constrained by mechanisms that are “flexible, deliberate, and implemented through cognitive control” (719). The paradigm here is the intentional generation of a deliberative process, or the intentional maintenance of attention on a task. The second dimension is characterized in terms of the degree to which thought is constrained by mechanisms that are automatic, in that they “operate outside of cognitive control to hold attention on a restricted set of information” (719). There are many ways thought may be automatically distracted—Christoff *et al.* mention affectively salient stimuli as one example.

They place mind wandering near other types of spontaneous thought in the two-dimensional space:

Within our framework, mind-wandering can be defined as a special case of spontaneous thought that tends to be more-deliberately constrained than dreaming, but less-deliberately constrained than creative thinking and goal-directed thought. In addition, mind-wandering can be clearly distinguished from rumination and other types of thought that are marked by a high degree of automatic constraints, such as obsessive thought. (719)

Now, this is not an explanation of why the mind wanders. It is, instead, a mapping of mind wandering onto a broader taxonomy of cognitive kinds, with special attention given to other modes of spontaneous thought. This taxonomy is useful for a number of reasons. For example, Christoff *et al.* map their taxonomy onto areas of the brain. So they say, e.g., that the part of the default network that centers on the medial temporal lobe is likely to be involved in the generation of mind wandering, as well as, via “its involvement in contextual associative processing” (724), the conceptual variability of some episodes of mind wandering. They also link the hippocampus to mind wandering, suggesting that it may contribute to the “imaginative construction” of hypothetical scenarios. Such mapping work from aspects of spontaneous thought onto activity patterns in large-scale brain networks affords fruitful suggestions for future study of the kinds of psychological patterns and activities that characterize mind wandering over time.

But there are possibilities and explanations that this approach does not (yet) address, and that potentially have consequences for the taxonomy of cognitive kinds that they offer.

Consider, e.g., the distinction they draw between mind wandering and creative thought. The difference is supposed to be a movement along the dimension of flexible, goal-directed constraints, with creative thinking requiring more cognitive control. But they note that creative thinking seems more complex than this:

Creative thinking may be unique among other spontaneous-thought processes because it may involve dynamic shifts between the two ends of the spectrum of constraints. The creative process tends to alternate between the generation of new ideas, which would be highly spontaneous, and the critical evaluation of these ideas, which could be as constrained as goal-directed thought in terms of deliberate constraints and is likely to be associated with a higher degree of automatic constraints than goal-directed thought because creative individuals frequently use their emotional and visceral reactions (colloquially often referred to as “gut” reactions) while evaluating their own creative ideas. (Box 1, 720)

I suggest that mind wandering is similarly complex. If the cognitive control proposal is correct, then in at least some cases mind wandering is initiated by processes of cognitive control, even though the goal driving mind wandering is not set explicitly by the agent. This could be captured by adding layers onto Christoff *et al.*’s taxonomy, deepening explanations of the etiology and function of each kind of spontaneous thought. And these deeper explanations at each place could be expected to bear fruit for understanding the dynamics of spontaneous thought. In particular, we might hope to find patterns in the neural dynamics that are predictive of the onset as well as the termination of mind wandering episodes, and that differentiate it from dreaming, creative thought, and perhaps from rumination. If the cognitive control proposal is correct, one task would be to map these patterns onto the expected value calculations the cognitive control system is performing. We would expect the dynamics of mind wandering to reflect the initiation of a search for a more rewarding goal, and to reflect attempts to make progress on this search. But now I’m jumping ahead, to predictions the proposal generates.

## Predictions

The cognitive control proposal makes predictions. Confirmation of these would be good news; disconfirmation would be bad news.

First, given the explanation offered for the initiation of mind wandering episodes, the proposal predicts that increases in reward for satisfying an occurrent goal would correlate with decreases in propensity to mind wander. It is well-confirmed that increasing reward leads to boosts in performance level, and to overcoming any purported “ego-depletion,” even for very boring tasks. Paradigms that have established this result could be used to test for the place of mind wandering in the behavioral data.

Second, the proposal predicts that increases in reward for non-occurrent goals the agent possesses would increase mind wandering. We have already seen that reminding agents of goals they possess, or of goals they will soon need to attempt to satisfy, leads to more mind wandering in the direction of these goals. The prediction here is more specific. If one were to, e.g., notify participants that they were soon to perform a task associated with some level of reward, and then to put participants through a low reward task, the prediction is that tendency to mind wander towards this task would be associated with the discrepancy in reward between the current and upcoming task.

Third, this proposal draws upon a view of the cognitive control system on which the learning of values associated with goals, and the learning of values associated with stimuli features predictive of goals, is crucial. So the proposal, plus plausible assumptions about reinforcement learning processes, predicts that it is possible to train participants to associate stimuli with certain goals, and that registration of such stimuli would generate mind wandering to the degree that the associated goal is rewarding. Very costly goals would produce little mind wandering. Cheap but rewarding goals would produce more.

And it may be possible to extend this result. It depends on what the agent associates with rewarding goals. Above I suggested that the system need not always compare value between explicit goals, and that the value computation might include an association between expected levels of reward and particular environments. If so, simply placing an agent in such environments would manipulate levels of unintentional mind wandering.

It may be useful to distinguish predictions this proposal makes from a related proposal: the current concerns hypothesis. The current concerns hypothesis (for which, see Klinger *et al.* 1973; Smallwood and Schooler 2006) has it that mind wandering is caused by a shift in salience—when one’s current goals (or concerns: here I use these terms interchangeably), become more salient than the external environment, one’s mind begins to wander. As Smallwood explains the view, “attention will be most likely to shift to self-generated material when such information offers larger incentive value than does the information in the external environment” (2013, 524). This proposal is distinct from mine in the following ways. First, I propose a specific mechanism, connected with recent modeling work in cognitive control, to explain the onset of mind wandering. Thus far, of course, the proposal can be seen as a specification of the current concerns hypothesis. Second, this mechanism initiates mind wandering not by turning attention to one’s current concerns, but by directed thought to search for a more valuable goal than the present one. So the cognitive control proposal makes predictions the current concerns hypothesis does not. For example, the cognitive control proposal predicts that propensity to mind wander could be increased by devaluing the present goal, independently of the salience of any of one’s current goals. That is, no matter how much one’s current goals or concerns lack salience, once could increase mind wandering by devaluing the occurrent goal. And it predicts that mind wandering will not turn directly to one’s other goals—the mind may wander to the environment, rather than to internal concerns, since this is one way the agent may attempt to find a more rewarding task. So we should, e.g., be able to find episodes of more intense environmental scanning as a part of the mind wandering episode. Indeed, if the environment is expected to contain valuable options, one would predict that this is where attention will go, rather than to any internal space of concerns.

This is not to deny that mind wandering represents a failure in some sense. McVay and Kane (2010b) have argued that mind wandering represents an executive control failure. What fails is a process of goal maintenance: “we suggest that goal maintenance is often hijacked by task-unrelated thought (TUT), resulting in both the subjective experience of mind wandering and habit-based errors” (324). The possibility I am raising is that failures of goal-maintenance could in another sense be successes of a different process. Indeed, perhaps processes of goal-maintenance are closely related to the value-based process of estimating the expected value of continuing on some task, or of searching for a new task, that I propose underlies unintentional mind wandering.

In sum, the proposal is plausible on its face. If correct, it promises to explain a range of data regarding mind wandering, and to explain the—from the agent’s conscious perspective very puzzling—initiation of mind wandering episodes. The proposal may also contribute to explanations of the dynamics of mind wandering. The predictions this proposal makes are testable, and work in this direction might take steps towards further integrating knowledge of how cognitive control works with knowledge of how mind wandering works.

## Philosophical Implications

I wish finally to relate this proposal to two leading philosophical accounts of mind wandering. Both of these accounts aim to capture mind wandering quite generally. I have noted in Mind wandering section that this is not my aim. Here, I want only to discuss implications for these more general accounts of mind wandering, if the cognitive control proposal about unintentional mind wandering is on track.

I turn first to Thomas Metzinger (2013). Metzinger’s thoughts on mind wandering are complex; here I focus only on one specific part of the picture, an item he calls mental autonomy. This is a technical notion for Metzinger, and he considers it essential: mind wandering, for Metzinger, essentially involves a loss of mental autonomy. He characterizes mental autonomy as:

[T]he ability to control the conscious contents of one’s mind in a goal-directed way, by means of attentional or cognitive agency. This ability can be a form of rational self-control, which is based on reasons, beliefs, and conceptual thought, but it does not have to be. What is crucial is the “veto component”: Being mentally autonomous means that all currently ongoing processes can in principle be suspended or terminated. This does not mean that they actually are terminated, it just means that the ability, the functional potential, is given and that the person has knowledge of this fact. M-autonomy is the capacity for causal self-determination on the mental level. (2013, 4)

I think the brush strokes Metzinger uses are too broad. I doubt we have veto control over every conscious process ongoing at a time. But I do think he locates an interesting phenomenon. In unintentional mind wandering, our knowledge (or awareness) that we might suspend, terminate, or re-direct aspects of the stream of consciousness lapses.

My question is this. Should we think of this lapse as the agent’s loss of control? As Metzinger has it, mind wandering essentially involves a lack of ability, and a lack of control—what he calls veto control. I agree that unintentional mind wandering does involve a loss of one kind of control. But I would underline the fact that there are multiple ways for a system to exercise control. Some of these involve consciousness in crucial ways. Some likely do not (Shepherd 2015). Knowledge that one can exercise control in some way at a moment can be useful. But a system may be well-designed, and exercise control in finding or executing goals, even if the system is not explicitly aware of processes that are performing these functions at a time.

Further, there are multiple ways for a system or an agent to possess an ability. The mind wandering agent may lack the ability to suspend, terminate, or re-direct elements of the stream of consciousness in virtue of her knowledge or awareness that she can do so. But she may retain the ability to suspend, terminate, or re-direct elements of the stream of consciousness in virtue of other features—perhaps in virtue of signals that emanate from the cognitive control processes I have emphasized.

This is not a merely verbal distinction. It is about how we understand the constitution of agency, and the kinds of properties that should be ascribed to mind wandering. If the cognitive control proposal is right, mind wandering emerges as an interesting case in which the seams of agency pull apart somewhat—we fail to notice that a non-conscious mechanism has turned the stream of consciousness in a different direction. But there may be good functional reasons for this operation, and it may contribute to an agent’s overall capacities to control the self in various environments and contexts.

I turn next to Zachary Irving (2016), who argues that mind wandering is unguided attention—or, as he says elsewhere (in Irving and Thompson 2019), unguided thinking. Irving seeks to preserve what he calls the “core intuition” that mind wandering is purposeless. He captures this intuition by explicating the sense in which mind wandering is unguided. It involves the absence of anything drawing the agent’s attention back to some goal.

An agent A’s attention is unguided if and only if A is not habitually guided to focus her attention on any information. In particular, she does not satisfy the counter-factual condition for attentional guidance: There is no information i such that, if A’s attention isn’t focused on i, she will notice, feel discomfited by, and thereby be disposed to correct this fact. (567)

I am not sure this is right. Mind wandering episodes are sometimes short. Sometimes they stop, it seems to me, precisely because we feel a sense that we were recently up to something, and we feel a pull to return. The cognitive control proposal might be able to explain this—one good move for the cognitive control system, in case of a failure to find a more rewarding task or goal, would be to return to the previous task.

Irving is aware that when it wanders, the mind frequently circles back to the agent’s goals. Does this not suggest guidance of some sort? Irving explains the tension by distinguishing between guidance and motivation. Motivated behavior only requires that an agent’s beliefs, desires, or goals are causal antecedents of the behavior. Guided behavior, by contrast, is explicated in terms of dynamics: it “involves the online monitoring and regulation of behavior” (563). Irving claims that mind wandering may be motivated, but it is not guided.

This aspect of Irving’s account does not compare favorably with the cognitive control proposal—if, of course, future work confirms the proposal. For Irving’s account offers no explanation of how causation by some belief or desire or goal helps explain how or why the wandering mind frequently turns to the agent’s goals. The cognitive control proposal has it that the wandering mind finds goals because that aim is what initiated and governs the mind wandering episode.

Further, if my proposal is right it is not entirely correct to think of mind wandering as unguided. It is, admittedly, not guided by any explicit intention the agent forms. In one sense of “guided,” then, Irving is right. But on the cognitive control proposal, mind wandering is a cognitive control process, and it does have a purpose. It seems purposeless to us in part because it is an interesting case in which some of the seams of agency pull apart somewhat—we do not notice that a non-conscious mechanism has turned the stream of consciousness in a different direction. And it seems purposeless to us in part because the course of the stream of consciousness during mind wandering is, as the cognitive control system plans it, meandering. It is meandering because the goal is to search, to explore, until a more rewarding task is found.

If these considerations are on track, we should say that mind wandering takes the form of a conscious but non-consciously guided process the aim of which is to find a rewarding goal or task. The connection with the cognitive control system explains the guidance aspect—the functionality of mind wandering—and affords the possibility of integration with work on the dynamics of mind wandering. The non-conscious aspect of the guidance explains the air of mystery surrounding mind wandering, why it seems purposeless, and why it seems to come about randomly.

## Conclusion

In this article, I have asked why the mind wanders. I focused on a sub-type of mind wandering—mind wandering that occurs independently of any reportable intention. I proposed that unintentional mind wandering is sometimes initiated and sustained by aspects of cognitive control. Unintentional mind wandering is caused by the cognitive control system precisely when, and because, the expected value of whatever the agent is doing—usually, exercising control towards achievement of some occurrent goal—is deemed too low, and this “too low” judgment generates a search for a better goal, or task.

This proposal generates testable predictions, and suggests open possibilities regarding the kinds of computations that may underlie unintentional mind wandering. My hope is that by connecting research on mind wandering with research on cognitive control resource allocation, fruitful strategies for modeling these computations may be taken from cognitive control research and deployed to help explain the initiation and dynamics of mind wandering episodes.

The cognitive control proposal also points us towards a fuller picture of human agency. In this picture, action control and intelligent thought are stitched together by conscious and non-conscious processes operating in concert. Future empirical work is critical to the confirmation of this picture, and to filling in the many unspecified details. This is so not least because, if the proposal I offer is on track, agents are not introspectively aware of the (good) rationale behind many mind-wandering episodes.
